# Muscularity-oriented disordered eating: investigating body image concerns and the moderating role of emotion dysregulation in cyclists

**DOI:** 10.1186/s40337-024-01109-6

**Published:** 2024-11-21

**Authors:** Jack Mazaraki, Kay Bussey, Mitchell Cunningham, Tom Jewell, Nora Trompeter

**Affiliations:** 1 Centre for Emotional Health, School of Psychological Sciences, Macquarie University, Sydney, Australia; 2 School of Psychology, The University of Sydney, Sydney, Australia; 3 Florence Nightingale Faculty of Nursing, Midwifery & Palliative Care, Kings College London, London, UK; 4 Psychological and Mental Health Services, Great Ormond Street Hospital NHS Foundation Trust, London, UK; 5 Population, Policy, and Practice Department, University College London, London, UK

**Keywords:** Cyclists, Muscularity-oriented eating disorders, Weight and shape concerns, Drive for leanness, Emotion dysregulation

## Abstract

A lean muscular body of low weight is often associated with performance for cyclists and can be difficult to achieve. Disordered eating for some cyclists may develop in the pursuit of lean muscularity which is perceived to be beneficial to performance. Further, emotion dysregulation may exacerbate negative feelings about body image, increasing the risk of developing disordered eating as a means of controlling weight and shape. While research in athlete populations has been substantial, disordered eating in cyclists is relatively under-explored. The current study examined whether shape and weight concern and drive for leanness were associated with muscularity-oriented disordered eating and whether these relationships were moderated by emotion dysregulation. Interactions between these factors were also investigated. Participants were 139 Australian competitive cyclists (112 males, 27 females, aged 18–68 years) who completed a self-report questionnaire comprised of measures of shape and weight concern, drive for leanness, muscularity-oriented eating and emotion dysregulation variables. Both shape and weight concern and drive for leanness had a unique association with muscularity-oriented disordered eating. Emotion dysregulation was found to have a unique association with muscularity-oriented disordered eating when controlling for drive for leanness, but not when shape and weight concern was controlled. Additionally, emotion dysregulation moderated the association between shape and weight concern and muscularity-oriented disordered eating, whereby the relationship was intensified at higher levels of emotion dysregulation. Our study has extended the literature by indicating that cyclists’ desire to attain a lean body is associated with muscularity-oriented disordered eating. Further, emotion dysregulation moderated this link, suggesting it could be a maintaining factor worth addressing.

**Supplementary Information:**

The online version contains supplementary material available at 10.1186/s40337-024-01109-6.

## Abstract (plain language summary)

Disordered eating is prevalent among athletes, especially those competing in endurance sports such as cycling. This study explored the relationship between body image concerns, emotion dysregulation and muscularity-oriented disordered eating (e.g., protein consumption and compensatory exercise) in cyclists. Results highlight how body dissatisfaction for cyclists is associated with the engagement of muscularity-oriented disordered eating. Additionally, the link between body image and muscularity-oriented disordered eating may be more pronounced amongst cyclists with higher emotion dysregulation.

**Supplementary Information:**

The online version contains supplementary material available at 10.1186/s40337-024-01109-6.

## Introduction

The physical demands of endurance sports such as long-distance running, swimming and road cycling create a scenario where a lean physique is advantageous for performance [1]. Consequently, endurance athletes may place an overvaluation on a lean body shape leading to a distorted sense of self-worth and contributing to the development of disordered eating [2]. Leanness is defined as toned, physically fit muscles with low body fat [3]. Prior research on athletes has demonstrated that endurance athletes perceive a greater pressure to be lean than non-endurance athletes, which is associated with higher levels of disordered eating [4]. Although a large amount of research into eating disorders in athlete populations has been completed [5], studies specifically focusing on cyclists and the influence of emotion dysregulation on their eating behaviours remain limited. Emotion dysregulation has been identified as an important variable that may influence the likelihood of engaging in disordered eating [6], but is yet to be studied in cyclists. For cyclists, there is a widespread belief in the sport that a lower body weight equates to better performance [7]. Additionally, cyclists are likely to be dissatisfied with their current body weight [8]. This desire for cyclists to maintain a high power-to-weight ratio by gaining musculature and efficiency as well as losing body weight, which is difficult to achieve, may further contribute to the development of disordered eating [9]. Cycling is an endurance sport that has been increasing in popularity. In Australia, across all forms of cycling, weekly participation has increased from 13.8% in 2019 to 18.0% in 2021 with 22.9% of males and 13.6% of females cycling [10]. The present study aims to investigate whether cyclists’ dissatisfaction with their bodies arises from feeling not thin or lean enough. To achieve this, the study will measure both concern about general body shape and weight, as well as a desire to become leaner. Among elite cyclists, research has demonstrated a risk of developing an eating disorder ranging from 57% to 59.9% [11, 12], with cyclists reporting higher levels of disordered eating compared to both other athletes and non-cyclists [13, 14]. Additionally, research involving cyclists within broader athlete populations consistently indicates a significant association between cycling and eating disorder symptoms, especially in competitive settings or when related to muscularity-oriented behaviours [15, 16]. However, one study comparing elite paddlers, runners, and cyclists found that paddlers were more likely to report eating disorder symptoms than cyclists [17]. Given the prevalence and significant impact of eating disorder behaviours among cyclists, particularly within competitive environments, further research is crucial to better understand the specific factors contributing to this risk and to inform prevention approaches and health promotion for this at-risk population.

Historically, the transdiagnostic model of eating disorders (CBT-E model; [2]) has focused on the presentation of pathological eating behaviours and symptoms among women [18], whereby, the development and maintenance of disordered eating focus on an excessive drive for thinness [19]. While this may capture traditional thinness-oriented disordered eating (i.e., fasting and fear of gaining weight), these criteria fail to accurately account for individuals whose eating pathology is centred around not being muscular or lean enough [20]. It has been demonstrated that muscularity-oriented disordered eating is relevant, particularly for men, as well as athletes who compete in sports where strength or power output is important [19, 21]. Muscularity-oriented disordered eating is characterised by an excessive focus on gaining muscle mass or achieving a highly muscular and lean physique [22]. Furthermore, Schofield and colleagues [23] found that road cyclists perceived leanness to be vital for success highlighting the importance of examining leanness and muscularity-oriented symptoms when researching the sport. Thus, for some cyclists, a desire to be lean may develop into and maintain unhealthy eating symptoms to achieve performance gains and an idealised lean body. Importantly, not all cyclists experience disordered eating. Therefore, it is pertinent to understand factors that may help explain why some (and not others) cyclists are at risk of experiencing disordered eating in relation to drive for leanness and shape and weight concern.

The CBT-E model proposes mood intolerance, an important aspect of emotion dysregulation, is a maintenance factor for disordered eating [2]. Whereby, a cycle of disordered eating can persist when individuals find it challenging to regulate their emotions and resort to disordered eating behaviours as coping mechanisms, particularly when those emotions stem from body dissatisfaction [2]. Emotion dysregulation refers to broader difficulties experienced when identifying one’s emotions and implementing adaptive regulatory strategies [24]. Additionally, empirical evidence has underscored the connection between emotion dysregulation and the eating disorder-specific risk factors of body dissatisfaction, dietary restraint and driven exercise [6]. Past research has identified all three of these factors as eating disorder symptoms among cyclists [7, 25, 26]. Hence, emotion dysregulation may be a key target for prevention in this potentially at-risk cohort. It has been reported as an important transdiagnostic correlate of eating disorders, whereby people may use unhealthy eating and exercise behaviours to cope with negative emotions [6, 27]. Additionally, previous research [28, 29] has demonstrated that emotion dysregulation was associated with muscle dysmorphia symptoms, highlighting the importance of studying emotion dysregulation in the context of muscularity-oriented disordered eating. Specifically, cyclists may experience body dissatisfaction in their pursuit of a lean physique. When coupled with poor emotional regulation skills, this combination may increase the likelihood of engaging in disordered eating, serving as a temporary escape or distraction from emotional distress related to body dissatisfaction. Of the sparse literature on the role of emotion dysregulation and disordered eating in athletes, Shriver and colleagues [30] found that female athletes who struggled to regulate their emotions had higher levels of disordered eating. Thus, cyclists with higher levels of emotion dysregulation may be at greater risk of developing disordered eating emanating from body image concerns.

## The current study

The factors which are associated with pathological eating behaviours and symptoms in cyclists are under-researched. Investigating these factors may provide useful information on eating disorder prevention efforts in cyclists, as well as health promotion to encourage cyclists to engage in a lifestyle that is less likely to produce negative health outcomes. As such, the current study aimed to address a key gap in the literature. Based on previous literature, four hypotheses were generated for muscularity-oriented disordered eating:

- H1a‐Shape and weight concern and emotion dysregulation will both have a unique association with muscularity-oriented eating scores.
- H1b‐Emotion dysregulation will moderate the association between shape and weight concern and muscularity-oriented eating scores, and these associations will be stronger at higher levels of emotion dysregulation.
- H2a‐Drive for leanness and emotion dysregulation will both have a unique association with muscularity-oriented eating scores.
- H2b‐Emotion dysregulation will moderate the association between drive for leanness and muscularity-oriented eating scores, and these associations will be stronger at higher levels of emotion dysregulation.

## Method

### Open science principles

Both the study design and data analysis plan were pre-registered (https://osf.io/jwzhg/?view_only=e92f3ffdfc054bf998d360989cd7bcc). All deviations from this plan are explicitly noted in the relevant sections below.

### Participants

Australian residents over the age of 18 years were eligible to participate. Participants were recruited through Australian cycling clubs, social media including Facebook cycling pages and groups, and the Macquarie University research participation pool. While we originally planned to compare the disordered eating levels and associations of interest between a cyclist and non-cyclist group, this was not possible due to significant differences in sociodemographic variables between the groups (differences between the two groups can be found in Supplementary 1).

Respondents included 112 men and 27 women aged between 18–68 years (*M* = 38.66, *SD* = 11.87). A post-hoc power analysis using the lowest effect size of the models (0.21) with an error probability rate of 0.05 and 139 participants indicated a power of 0.99 (G*Power; [31]). Thus, the obtained sample size of 139 was adequate to test the study’s hypotheses. Of the cyclist respondents 21 (15.1%) were recruited through the Macquarie University research pool and 118 (84.9%) were recruited from community outlets. The level of events in which the cyclists competed were World (n = 4, 2.9%), National (n = 13, 9.3%), State (n = 21, 15.1%), Local (n = 61, 43.8%), and Casual (n = 40, 28.7%) riders. The sample consisted predominantly of competitive riders (local level and above) who rode in organised cycling events. All participants cycled for health and training purposes and did not cycle purely as a means of transportation (i.e., commuting to work). On average per week, cyclists rode for 10 h and completed 90 min of resistance training.

### Measures

#### Shape and weight concern

Participants’ shape and weight concern were assessed using the combined Shape Concern and Weight Concern subscales of the EDE-Q [32]. Participants were asked to rate the frequency/severity of their body image concerns (e.g., “Have you had a definite desire to have a totally flat stomach?”) on a 7-point response scale (0 = *No days/Not at all* to 6 = *Everyday/Markedly*). Previous factor analyses indicated that the two subscales can be combined into one factor due to their substantial overlap [33]. The combined measure includes 12 items regarding body image concerns over the past 28 days. Items on the subscale were averaged to provide a total score across the 12 items, whereby higher scores indicate greater shape and weight concern. While the subscale has demonstrated good internal consistency in community samples [34], it has yet to be validated in an athlete sample. In the present study, the scale demonstrated excellent internal consistency (*α* = 0.93).

#### Drive for leanness

Participants’ drive for leanness were evaluated with the 6-item Drive for Leanness Scale [3]. The scales items (e.g., “It is important to have well-defined abs”) were measured on a 6-point response scale (1 = *Never* to 6 = *Always*). Higher mean scores indicate a greater drive for leanness. Evidence supports good internal consistency in community samples [3], and athletes [35]. In the present study, the scale demonstrated good internal consistency (*α* = 0.87).

#### Emotion dysregulation

Emotion dysregulation was measured using the Difficulties in Emotion Regulation Scale—Short Form [36]. The measure is a shortened 18-item version of the original Difficulties in Emotion Regulation Scale [37]. As with the original scale, the measure assesses six factors of emotion dysregulation, those being nonacceptance of emotional responses (e.g., “When I’m upset, I become embarrassed for feeling that way”), difficulty engaging in goal-directed behaviour (e.g., “when I’m upset, I have difficulty getting work done”), impulse control difficulties (e.g., “when I’m upset, I become out of control”), emotional awareness (e.g., “When I’m upset, I acknowledge my emotions “), limited access to emotion regulation strategies (e.g., “When I’m upset, it takes me a long time to feel better”), and lack of emotional clarity (e.g., “I am confused about how I feel”). Participants rated how often the statements apply to them on a 5-point response scale (1 = *Almost Never* to 5 = *Almost Always*). A total score was obtained from a mean score of all items, where higher scores indicate greater difficulties in emotion regulation. Evidence supports good internal consistency in community samples [36], and athletes [38]. In the present study, the scale demonstrated good internal consistency (*α* = 0.89).

#### Muscularity oriented disordered eating

Participants’ attitudes and behaviours concerning leanness and muscularity-oriented eating were assessed with the 27-item Eating for Muscularity scale [39]. The Eating for Muscularity scale assesses eating disorder symptoms that are motivated by the pursuit of a lean, muscular body ideal. Participants were asked to rate the frequency/severity of their eating for muscularity symptoms (e.g., “I have used dieting methods to become more muscular”) on a 7-point response scale (0 = *No days/Not at all* to 6 = *Everyday/Markedly*). A total score was obtained by averaging all items, with higher scores indicating greater muscularity-oriented disordered eating. The Eating for Muscularity scale has demonstrated high internal consistency [39], but has not yet been validated in an athlete population. In the present study, the scale demonstrated excellent internal consistency (*α* = 0.92).

### Procedure

The project was approved by the authors’ University Human Research Ethics Committee. All study materials were completed through Qualtrics [40]. Participants provided informed consent and confirmed eligibility before responding to demographic questions and the study measures. The completion time for the survey was approximately 15 min. Participants entered a raffle draw for one of three $50 gift vouchers. To ensure data quality 159 suspected bot respondents were screened and excluded from the survey.

### Data analytic plan

Analyses were conducted with STATA version 17. Due to significant differences in sociodemographic variables between the cyclists and non-cyclists, we proceeded by focusing on the cyclist group and did not examine the Multivariate Analysis of Covariance to examine group differences, as was presented in the pre-registration. Additionally, our pre-registered analysis included utilising two items from the Restraint subscale of the EDE-Q [32] to explore thinness-oriented alongside muscularity-oriented disordered eating. However, in the peer review process, it was recommended this measure be abandoned as the items do not exclusively measure thinness-oriented eating behaviours (and may also be relevant to muscularity-oriented goals). We agree with this position and proceeded with muscularity-oriented disordered eating as the primary (and only) outcome variable. For completeness based on our preregistered analytic plan, we report findings from the regression analysis predicting thinness-oriented disordered eating in Supplementary 1.

Due to their association with eating disorder characteristics, gender, age, and body mass index (BMI) were controlled for [41] Further, while examining gender differences of eating disorder behaviour in cyclists is out of the scope of this study, a sensitivity analysis was conducted to assess the effect gender contributes to each model (see supplementary 1). For Hypotheses 1a – 2b, to examine the unique association between shape and weight concern and drive for leanness with emotion dysregulation on muscularity-oriented eating scores, two separate hierarchical regression analyses were conducted. For each regression model, the control variables (gender, age, and BMI) were entered in step 1. Secondly, emotion dysregulation and shape and weight concern/drive for leanness were entered in step 2. Finally, the interaction term (emotion dysregulation x shape and weight concern/drive for leanness) was added in step 3. To control for multiple comparisons, the Benjamini–Hochberg procedure was applied to the multiple regression analysis, with a paper-wide false discovery rate of 0.05, resulting in a critical alpha of 0.022.

First, descriptive statistics were calculated for the variables of interest. Assumptions were then tested to assess the distributions of the variables. While the assumption for multicollinearity was met (*VIF* = 1.53 Shape and Weight Concern model; *VIF* = 1.00 Drive for Leanness model), the assumption of normality was not met. We used Spearman’s correlation analysis, as well as bias-corrected bootstrapping based on 1000 resamples for both regression models to account for these violations. Bootstrapping analyses were carried out to provide 95% confidence intervals. This approach has been argued to provide robust results when the assumption of normality is violated and is considered more favourable compared to other methods like transformations or relying on the robustness of F-tests and t-tests [42]. Significant interaction terms were probed using simple slope analysis at low (− 1 SD), average and high levels (+ 1 SD) of difficulties of emotion regulation scale scores.

Five per cent of participants’ responses were missing and were considered missing at random (Little MCAR test: *χ2*(44) = 51.05, *p* = 0.216). Therefore, listwise deletion was applied across analyses [43].

## Results

### Sample characteristics

Means and standard deviations of measures among cyclists are presented in Table 1. In the current study, 26% of cyclists scored above the EDE-Q global gender unique clinical cutoff (> 2.3 for women and > 1.68 for men; [44, 45]) for likelihood of having an eating disorder.

**Table 1:** Means and standard deviations of demographic variables and study measures

Variables	M (SD)
Gender (% female)	19.4
Age (in years)	38.66 (11.87)
Body mass index	24.58 (3.93)
Shape and weight concern	1.66 (1.31)
Drive for leanness	3.57 (0.70)
Emotion dysregulation	2.08 (0.56)
Muscularity-oriented eating	0.90 (0.84)

### Correlations

Table 2 presents the correlations between study variables. Muscularity-oriented eating scores had a significant positive association with shape and weight concern, drive for leanness and emotion dysregulation.

**Table 2:** Spearman’s correlations between key study variables

	1	2	3	4	5	6
1. Shape and weight concern	–					
2. Drive for leanness	0.26**	–				
3. Emotion Dysregulation	0.30**	− 0.03	–			
4. Muscularity-oriented eating	0.48**	0.47**	0.22*	–		
5. Age (in years)	− 0.01	− 0.07	− 0.22*	− 0.02	–	
6. Body Mass Index	0.26**	0.01	− 0.19	− 0.04	0.02	–

Significant correlations are bolded. *p < 0.05, ** *p* < 0.01

### Regression analyses

#### Muscularity-oriented eating (outcome criterion): associations with shape and weight concern and emotion dysregulation

As can be seen in Table 3, shape and weight concern, but not emotion dysregulation, had a unique association with muscularity-oriented eating scores, whereby higher levels of shape and weight concern were associated with higher levels of muscularity-oriented eating scores. Thus, Hypothesis 1a was partially supported.

**Table 3:** Muscularity-oriented eating scores regressed on shape and weight concern and emotion dysregulation for cyclists

	Muscularity-oriented eating scores		
	β	S.E	95% CI
Step 1			
Age	− 0.01	0.01	[− 0.02, 0.01]
Gender	0.24	0.19	[− 0.12, 0.60]
BMI	− 0.01	− 0.02	[− 0.04, 0.03]
${R}^{2}$ adjusted	0.010		
Step 2			
Shape and Weight Concern	0.40	0.07	[0.25,0 0.51]**
Emotion Dysregulation	0.15	0.14	[− 0.12,0 0.42]
${R}^{2}$ change	0.360		
Step 3			
SWC x Emotion Dysregulation	0.18	0.07	[0.00, 0.30]*
${R}^{2}$ change	0.041		
Total ${R}^{2}$ adjusted	0.401		

SWC, Shape and weight concern; CI, Confidence Interval; Bias corrected beta coefficients are reported (β). Bootstrap standard error (S.E.). Bootstrap bias-corrected confidence values are reported. Reference category for gender was ‘Male’. Benjamini- Hochberg corrected critical value = 0.022 Significant associations are bolded.*p < 0.022, **p < 0.01

**Fig. 1:**
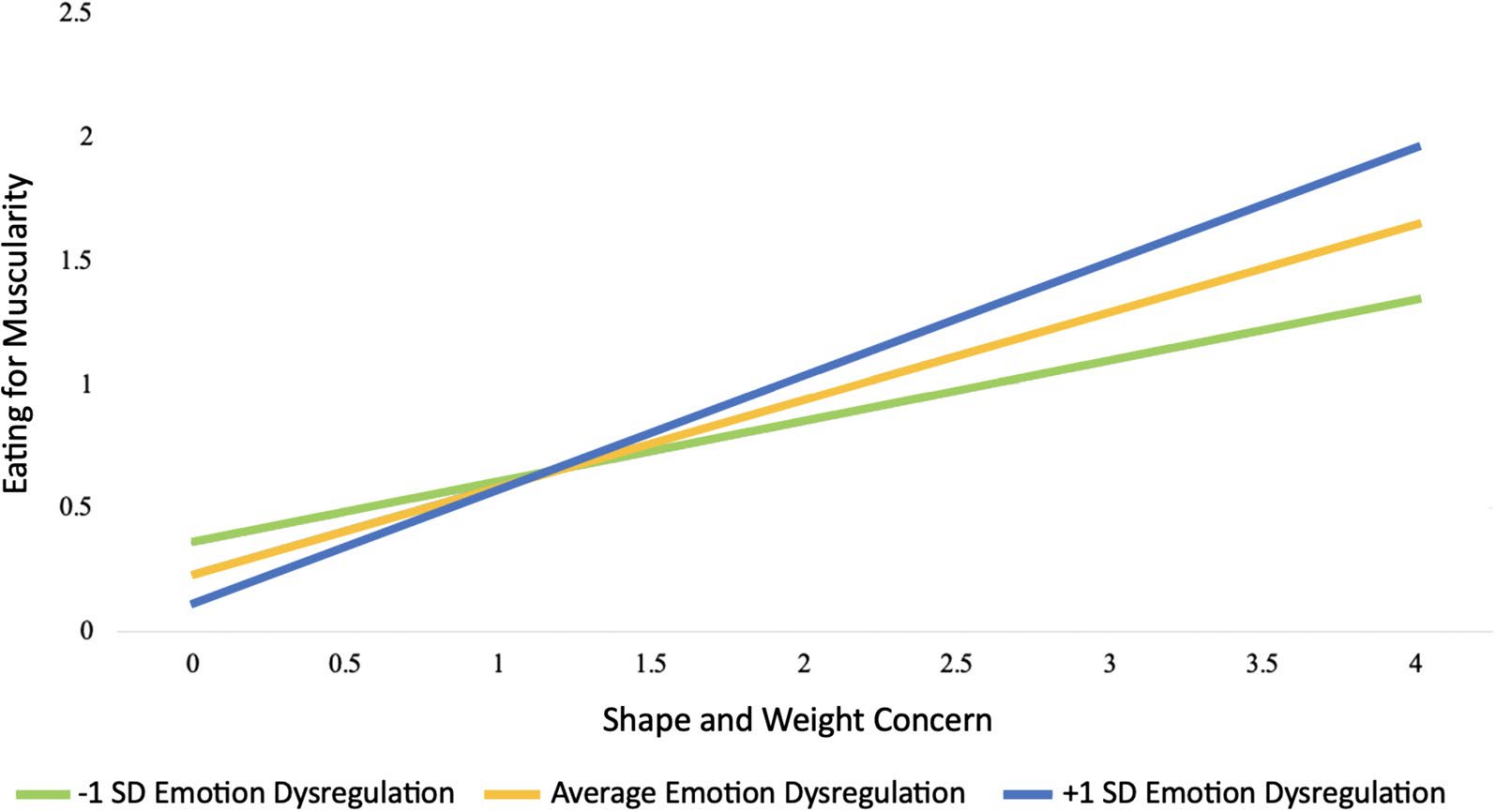
Moderation of the effect of shape and weight concern and emotion dysregulation on muscularity-oriented eating scores. *Note* Muscularity-oriented eating scores and shape and weight concern scales are from 0 (low) – 6 (high)

Additionally, to test hypothesis H1b, the significance of the interaction term between emotion dysregulation and shape and weight concern on muscularity-oriented eating scores was assessed. The results revealed that the interaction term between emotion dysregulation and shape and weight concern emerged as significant, whereby emotion dysregulation intensified the relationship. Analysis of simple slopes revealed that the association between shape and weight concern and muscularity-oriented eating scores became stronger as difficulty in emotion regulation scores increased. These results support hypothesis 1b demonstrating an increase in the relationship between shape and weight concern and muscularity-oriented eating scores from low levels (β = 0.64, *p* = 0.007), to average levels (β = 0.74, *p* < 0.001), and most strongly at high levels (β = 0.84, *p* < 0.001) of emotion dysregulation (see Fig. 1).

#### Muscularity-oriented eating (outcome criterion): associations with drive for leanness and emotion dysregulation

As can be seen in Table 4, both drive for leanness and emotion dysregulation had a unique association with muscularity-oriented eating scores, whereby both higher levels of drive for leanness and emotion dysregulation, were associated with higher levels of muscularity-oriented eating scores. Thus, Hypothesis 2a was fully supported.

**Table 4:** Muscularity-oriented eating scores regressed on drive for leanness and emotion dysregulation for cyclists

	Muscularity-oriented eating scores		
	β	S.E	95% CI
Step 1			
Age	− 0.01	0.01	[− 0.02, 0.01]
Gender	0.24	0.19	[− 0.12, 0.60]
BMI	− 0.01	− 0.02	[− 0.04, 0.03]
${R}^{2}$ adjusted	0.010		
Step 2			
Drive for leanness	0.38	0.09	[0.21, 0.55]*
Emotion dysregulation	0.50	0.13	[0.26, 0.76]*
${R}^{2}$ change	0.205		
Step 3			
DFL x emotion dysregulation	0.07	0.18	[− 0.20, 0.52]
${R}^{2}$ change	− 0.005		
Total ${R}^{2}$ adjusted	**0.210**		

DFL, drive for leanness; CI, confidence interval; Bias corrected beta coefficients are reported (β). Bootstrap standard error (S.E.). Bootstrap bias-corrected confidence values are reported. Reference category for gender was ‘Male’. Benjamini- Hochberg corrected critical value = 0.022. Significant associations are bolded. *p < 0.022

Additionally, to test Hypothesis 2b, the significance of the interaction term between emotion dysregulation and drive for leanness on muscularity-oriented eating was assessed.

The results revealed that the interaction was not significant and therefore Hypothesis 2b was not supported.

## Discussion

The present study investigated the relationships between both drive for leanness and shape and weight concern with muscularity-oriented disordered eating for cyclists, and whether these were moderated by emotion dysregulation. Given that existing research on disordered eating in cycling has often focused on broader eating disorder symptoms, there remains a significant gap in understanding the specific role of muscularity-oriented eating disorders and how factors like emotion dysregulation contribute to these behaviours in cyclists.

The hypotheses that shape and weight concern, drive for leanness, and emotion dysregulation would be uniquely associated with muscularity-oriented disordered eating were partially supported. Shape and weight concern and drive for leanness both had unique positive associations with muscularity-oriented eating scores. Whereas, emotion dysregulation was found to only have a unique positive association with muscularity-oriented eating when drive for leanness was held constant but not shape and weight concern. This finding builds upon Byrne and Mclean’s [4] research on endurance athletes, which revealed that pressure to be lean and general shape and weight concern were associated with higher levels of disordered eating. It highlights that dissatisfaction with one’s body, whether arising from feeling insufficiently thin or lean, is correlated with the engagement of muscularity-oriented disordered eating among cyclists.

Further, the present study sought to examine the moderating effect of emotion dysregulation with shape and weight concern regarding muscularity-oriented disordered eating. Findings demonstrated that emotion dysregulation moderated the association between shape and weight concern and muscularity-oriented eating. Specifically, the strongest association between shape and weight concern and muscularity-oriented eating scores was observed among cyclists with the highest levels of emotion dysregulation. This highlights that emotion dysregulation and shape and weight concern may have a multiplicative effect on muscularity-oriented eating scores and represents a novel finding for engagement in muscularity-oriented disordered eating. Additionally, variations in the moderating effect of emotion dysregulation and shape and weight concern, as opposed to drive for leanness, could be attributed to disparities in the focal points of measurement. The drive for leanness scale evaluates body image ideals (e.g., "I think the best looking bodies are well-toned"), whereas specific questions of the shape and weight concern scale gauge negative emotions linked to body image worries (e.g., "How uncomfortable have you felt seeing your body?"). These results are in line with dimensional frameworks of psychopathology that suggest symptoms arise from a combination of syndrome-specific moderators and transdiagnostic risk factors, in this case, shape and weight concern and emotion dysregulation [46, 47]. Thus, cyclists experiencing body image concerns and related negative affect, when coupled with poor emotion regulation, are more likely to engage in muscularity-oriented disordered eating. These findings support Racine et al. [48] who established that among adolescent and young women, disordered eating (i.e., body dissatisfaction, restricted eating) moderated the association between the transdiagnostic factor of negative urgency (impulsive behaviour in response to distress, which has been closely linked to emotion dysregulation; [49]) and binge eating. Furthermore, these findings extend upon prior research by Murray et al. [28] and Murray et al. [29] that demonstrated difficulty in emotion regulation was related to muscularity-oriented disordered eating, highlighting that high levels of body dissatisfaction may intensify the relationship.

The combined shape and weight concern scale measured in this study broadly encompasses multiple aspects of shape- and weight-related attitudes, including an over-valuation of shape and weight, and fear of weight gain [32]. In contrast, drive for leanness does not emphasize extreme thinness or muscularity exclusively and instead represents an athletic body composition that is low in body fat with toned muscles [3]. In the present study, differences in the association of emotion dysregulation and disordered eating, when controlling for shape and weight concern or drive for leanness, may suggest that shape and weight concern and emotion dysregulation may explain similar variance in muscularity-oriented disorder eating. Further, it may also suggest that a lean body ideal is not related to emotion dysregulation when explaining disordered eating. There appear to be two potential reasons for this finding. First, negative emotions that arise for cyclists may stem more from body dissatisfaction that is oriented towards not being thin enough rather than not being lean enough. Thus, explaining the unique variance that arose for emotion dysregulation and drive for leanness. However, this is contrary to prior findings that demonstrated individuals who have a desire for a lean muscular body show the same risk of exhibiting unhealthy behavioural (i.e., compulsive exercise, fasting) and psychological (i.e., negative emotional states) outcomes as those with a desire to be thin [50]. Secondly, questions asked in the Shape and Weight Concern scale explicitly assess the relationship between body dissatisfaction and negative emotions (i.e., “How uncomfortable have you felt seeing your body?”). On the other hand, questions asked in the Drive for Leanness scale are more oriented towards lean body ideal preferences (i.e., “I think the best looking bodies are well-toned”) and not how an inability to attain such ideals influences emotions. Therefore, disparities in how the two separate constructs are measured may instead be influencing why their relationship with emotion dysregulation differs. Ultimately, however, these results highlight that a drive for a lean muscular body may play a role in the domain of eating disorder psychopathology, particularly for athletes.

The findings from the present study may have important implications for prevention and intervention programs aimed at reducing disordered eating among cyclists. First, our study extends upon prior disordered eating research as well as the CBT-E model indicating that in addition to shape and weight concern and emotion dysregulation, well-established disorder-specific risk factors [2], drive for leanness may play an important role in the engagement of muscularity-oriented disordered eating for road cyclists. In pursuit of performance increases, cyclists may engage in training methods (i.e., exercise, carbohydrate restriction) that can indistinguishably turn into disordered eating. As argued in previous research on disordered eating in athletes, certain training and eating behaviours may reflect a rational response to achieving a lean body composition rather than psychopathology [51]. However, with this lean ideal regularly being portrayed in society and the sport as a benchmark of physical health and peak performance it is important that cyclists are aware of the potential risks of having a desire for a lean body [52]. This is especially true when feelings of body dissatisfaction are motivating the desire to alter body shape and size. Furthermore, it is important that cyclists and coaches are cognisant of when disordered eating is being used to deal with negative emotions that arise from feelings of body dissatisfaction or other facets of life. Therefore, prevention programs for cyclists may benefit from targeting these variables, focusing on the development of better emotional coping skills and promoting positive body image in the sport.

Additionally, the current study has important theoretical implications. Traditionally, etiological models of the psychopathology of eating disorders, such as the CBT-E model, have focussed on a thinness-oriented body ideal that underpins disordered eating [2]. However, in line with recent research [20], the present study demonstrated how leanness and muscularity-oriented body ideals may also underpin disordered eating in cyclists. These results provide further evidence for the need to modify traditional eating disorder conceptualisations and prevention approaches to better address and mitigate eating disorder pathology that is oriented towards leanness and muscularity. Furthermore, our findings exhibit how when body dissatisfaction is coupled with poor emotion regulation it may increase the likelihood of engaging in muscularity-oriented disordered eating. Thus, enhancing the emotion regulation skills of cyclists may aid in effectively navigating negative emotions stemming from body image concerns, promoting a more adaptive response rather than resorting to disordered eating behaviours. Therefore, the present findings suggest that muscularity-oriented eating symptoms may have similar developmental and maintenance factors as traditional thinness-oriented symptoms and warrant further investigation.

While the present study had several strengths, notably implementing a new muscularity-oriented measurement scale that expands beyond conventional thinness-oriented conceptualisations used in most studies to examine disordered eating, several limitations should also be considered. First, the current study relied on cross-sectional data. Albeit the variables in the study displayed significant associations with each other, no inferences can be made regarding the temporal and casual nature of the relationships studied here. Furthermore, the global indicator of the Difficulties in Emotion Regulation Scale—Short Form was utilised and not the sub-scales (i.e., non-acceptance of emotional responses, lack of emotional awareness). As such, future research should look at the sub-scales to identify specific moderator relations between shape and weight concern and muscularity-oriented disordered eating for cyclists. Additionally, due to the necessity to omit the non-cyclist control group that arose from significant differences in sociodemographic variables, we were unable to determine how the relationships evidenced here differed between cyclists and non-cyclists. It is important then that future research investigates if selection effects were present in the current study. This may allow for more accurate information on how prevention and education programs are utilised to encourage cyclists to engage in a lifestyle and training that is less likely to produce negative health outcomes. Furthermore, the changes in regression outcomes when female cyclists were excluded in the sensitivity analysis—particularly the loss of significance in the interaction between shape and weight concern and emotion dysregulation on muscularity-oriented eating—underscores the necessity of examining gender differences in future research. To gain a deeper understanding of the distinct pathways and effects in male and female populations, future studies should incorporate interaction terms between gender and key predictors and consider using gender-specific models. However, it is possible that in this study differences in results might also be attributable to the reduced sample size after excluding female participants. The smaller sample may have lacked the statistical power needed to detect significant effects that were evident in the combined analysis.

In conclusion, this study advances the current literature by providing an examination of the leanness and muscularity-oriented disordered eating of cyclists. Cyclists represent an understudied population who are potentially at increased likelihood of engaging in disordered eating. These findings highlight how body dissatisfaction and the pursuit of a lean body may lead to engagement in muscularity-oriented disordered eating. Additionally, these results demonstrate how prevention programs may benefit from educating cyclists on emotion regulation skills, especially those with higher levels of shape and weight concern. Furthermore, the results offer important insight into how shape and weight concern, drive for leanness, and emotion dysregulation are associated with leanness- and muscularity-oriented disordered eating in cyclists.

## Supplementary Information

Supplementary file 1.

## Data Availability

Deidentified data are available upon request from the lead author (J.M.), pertaining to approval from the author’s institutional ethics committee.
