# Supplementary material for: Muscularity-oriented disordered eating: investigating body image concerns and the moderating role of emotion dysregulation in cyclists
Source: J Eat Disord. 2024 Nov 21;12:189. doi: 10.1186/s40337-024-01109-6 (PMC11580638; doi:10.1186/s40337-024-01109-6)
Supplement: Supplementary file 1 [file 40337_2024_1109_MOESM1_ESM.docx]

Supplementary 1

*Means and Standard Deviations of Scores on Demographic and Psychological Factors for Cyclists and Non-Cyclists*

| Variables | Cyclist sample (*n* = 139) | Non-cyclist sample (*n* = 161) |
| --- | --- | --- |
| Age (in years) | 38.66 (11.87)* | 25.60 (10.28)* |
| Body Mass Index | 24.58 (3.93) | 24.20 (5.14) |
| Socioeconomic status (decile) | 7.22 (2.60) | 7.63 (2.88) |
| Shape and weight concern | 1.66 (1.31)* | 2.12 (1.55)* |
| Drive for leanness | 3.57 (.70)* | 3.16 (.80)* |
| Emotion dysregulation | 2.08 (.56)* | 2.35 (.70)* |
| EDE-Q total | 1.46 (1.17)* | 1.88 (1.40)* |
| Thinness-oriented eating | 1.51 (1.42) | 1.67 (1.67) |
| Muscularity-oriented eating | 0.90 (.84) | 0.98 (.80) |
|  | *N (%)* | |
| Gender (% female) | 19.4* | 70.8* |
| Females EDE-Q total > 2.3 | 40.0 | 40.5 |
| Males EDE-Q total > 1.68 | 27.7 | 24.4 |

*Note.* EDE-Q = Eating Disorder Examination Questionnaire. Significant differences are indicated *p < .05.

Supplementary 2

*Thinness-Oriented Eating Scores Regressed on Shape and Weight Concern and Emotion Dysregulation for Cyclists*

|  | Thinness-Oriented Eating Scores | | |
| --- | --- | --- | --- |
|  | β | σ | 95% CI |
| Step 1 |  |  |  |
| Age | .01 | .01 | [-.03, .02] |
| Gender | .44 | .38 | [-.28, 1.14] |
| BMI | .02 | .03 | [-.03, .08] |
| $R^{2}$ adjusted | .000 |  |  |
| Step 2 |  |  |  |
| Shape and Weight Concern | .71 | .09 | **[.54, .89]**** |
| Emotion Dysregulation | .00 | .25 | [-.42, .52] |
| $R^{2}$ change | .343 |  |  |
| Step 3 |  |  |  |
| SWC x Emotion Dysregulation | .06 | .19 | [-.25, .37] |
| $R^{2}$ change | .000 |  |  |
| Total $R^{2}$ adjusted | .343 |  |  |

*Note*. Shape and weight concern = SWC; Confidence Interval = CI; Bias corrected beta coefficients are reported (β). Bootstrap standard error (σ). Bootstrap bias-corrected confidence values are reported. Reference category for gender was ‘Male’. Significant associations are bolded. *p < .05, **p < .01

Supplementary 3

*Thinness-Oriented Eating Scores Regressed on Drive for Leanness and Emotion Dysregulation for Cyclists*

|  | Thinness-Oriented Eating Scores | | |
| --- | --- | --- | --- |
|  | β | σ | 95% CI |
| Step 2 |  |  |  |
| Drive For Leanness | .66 | .18 | **[.32, 1.03]**** |
| Emotion Dysregulation | .70 | .25 | **[.16, 1.20]*** |
| $R^{2}$ change | .144 |  |  |
| Step 3 |  |  |  |
| DFL x Emotion Dysregulation | .41 | .37 | [-.31, 1.15] |
| $R^{2}$ change | .004 |  |  |
| Total $R^{2}$ adjusted | **.148** |  |  |

*Note*. DFL = Drive for Leanness; Confidence Interval = CI; Bias corrected beta coefficients are reported (β). Bootstrap standard error (σ). Bootstrap bias-corrected confidence values are reported. Reference category for gender was ‘Male’. Significant associations are bolded. *p < .05, **p < .01

Supplementary 4

*Means and Standard Deviations of Demographic Variables and Study Measures for Males and Females*

|  | *Male* | *Females* |
| --- | --- | --- |
| Variables | *M* (*SD*) | *M* (*SD*) |
| Number of Respondents | 112 | 27 |
| Age (in years) | 39.07 (12.08) | 37.07 (10.79) |
| Body Mass Index | 24.85 (4.09) | 23.49 (2.89) |
| Shape and weight concern | 1.39 (1.12) | 2.62 (1.53) |
| Drive for leanness | 3.52 (.72) | 3.74 (.63) |
| Emotion dysregulation | 2.04 (.52) | 2.24 (.71) |
| Muscularity-oriented eating | .84 (.81) | 1.11 (.91) |

*Muscularity-Oriented Eating Scores Regressed on Shape and Weight Concern and Emotion Dysregulation for Male Cyclists*

|  | Muscularity-Oriented Eating Scores | | |
| --- | --- | --- | --- |
|  | β | S.E. | 95% CI |
| Step 1 |  |  |  |
| Age | .00 | .01 | [-.02, .01] |
| BMI | .01 | .01 | [-.04, .04] |
| $R^{2}$ adjusted | -.010 |  |  |
| Step 2 |  |  |  |
| Shape and Weight Concern | .42 | .08 | **[.23, .55]**** |
| Emotion Dysregulation | -.08 | .14 | [-.36, .20] |
| $R^{2}$ change | .288 |  |  |
| Step 3 |  |  |  |
| SWC x Emotion Dysregulation | .04 | .17 | [-.24, .41] |
| $R^{2}$ change | -.007 |  |  |
| Total $R^{2}$ adjusted | .271 |  |  |

*Note*. Shape and weight concern = SWC; Confidence Interval = CI; Bias corrected beta coefficients are reported (β). Bootstrap standard error (S.E.). Bootstrap bias-corrected confidence values are reported. Significant associations are bolded. *p < .05, **p < .01

*Muscularity-Oriented Eating Scores Regressed on Drive for Leanness and Emotion Dysregulation for Male Cyclists*

|  | Muscularity-Oriented Eating Scores | | |
| --- | --- | --- | --- |
|  | β | S.E. | 95% CI |
| Step 1 |  |  |  |
| Age | .00 | .01 | [-.02, .01] |
| BMI | .01 | .01 | [-.04, .04] |
| $R^{2}$ adjusted | -.010 |  |  |
| Step 2 |  |  |  |
| Drive For Leanness | .45 | .09 | **[.29, .66]**** |
| Emotion Dysregulation | .25 | .13 | **[.01, .50]*** |
| $R^{2}$ change | .185 |  |  |
| Step 3 |  |  |  |
| DFL x Emotion Dysregulation | .15 | .20 | [-.16, .60] |
| $R^{2}$ change | -.005 |  |  |
| Total $R^{2}$ adjusted | .170 |  |  |

*Note*. DFL = Drive for Leanness; Confidence Interval = CI; Bias corrected beta coefficients are reported (β). Bootstrap standard error (S.E.). Bootstrap bias-corrected confidence values are reported. Significant associations are bolded. *p < .05, **p < .01

*Muscularity-Oriented Eating Scores Regressed on Shape and Weight Concern and Emotion Dysregulation for Female Cyclists*

|  | Muscularity-Oriented Eating Scores | | |
| --- | --- | --- | --- |
|  | β | S.E. | 95% CI |
| Step 1 |  |  |  |
| Age | -.03 | .02 | [-.07, .01] |
| BMI | -.04 | .06 | [-.16, .07] |
| $R^{2}$ adjusted | .039 |  |  |
| Step 2 |  |  |  |
| Shape and Weight Concern | .30 | .09 | **[.07, .46]**** |
| Emotion Dysregulation | .66 | .23 | **[.21, 1.12]*** |
| $R^{2}$ change | .668 |  |  |
| Step 3 |  |  |  |
| SWC x Emotion Dysregulation | .22 | .13 | [-.08, .45]* |
| $R^{2}$ change | .045 |  |  |
| Total $R^{2}$ adjusted | .755 |  |  |

*Note*. Shape and weight concern = SWC; Confidence Interval = CI; Bias corrected beta coefficients are reported (β). Bootstrap standard error (S.E.). Bootstrap bias-corrected confidence values are reported. Significant associations are bolded. *p < .05, **p < .01

*Muscularity-Oriented Eating Scores Regressed on Drive for Leanness and Emotion Dysregulation for Female Cyclists*

|  | Muscularity-Oriented Eating Scores | | |
| --- | --- | --- | --- |
|  | β | S.E. | 95% CI |
| Step 1 |  |  |  |
| Age | -.03 | .02 | [-.07, .01] |
| BMI | -.04 | .06 | [-.16, .07] |
| $R^{2}$ adjusted | .039 |  |  |
| Step 2 |  |  |  |
| Drive For Leanness | .31 | .26 | [-.32, .69] |
| Emotion Dysregulation | 1.04 | .21 | **[.55, 1.39]**** |
| $R^{2}$ change | .516 |  |  |
| Step 3 |  |  |  |
| DFL x Emotion Dysregulation | -.05 | .45 | [-.92, 92] |
| $R^{2}$ change | -.020 |  |  |
| Total $R^{2}$ adjusted | .537 |  |  |

*Note*. DFL = Drive for Leanness; Confidence Interval = CI; Bias corrected beta coefficients are reported (β). Bootstrap standard error (S.E.). Bootstrap bias-corrected confidence values are reported. Significant associations are bolded. *p < .05, **p < .01
